# Elevated Circulating Levels and Tissue Expression of Pentraxin 3 in Uremia: A Reflection of Endothelial Dysfunction

**DOI:** 10.1371/journal.pone.0063493

**Published:** 2013-05-03

**Authors:** Anna Witasp, Mikael Rydén, Juan Jesús Carrero, Abdul Rashid Qureshi, Louise Nordfors, Erik Näslund, Folke Hammarqvist, Samsul Arefin, Karolina Kublickiene, Peter Stenvinkel

**Affiliations:** 1 Divisions of Renal Medicine and Baxter Novum, Department of Clinical Science, Intervention and Technology, Karolinska Institutet, Stockholm, Sweden; 2 Department of Medicine (H7), Karolinska Institutet, Stockholm, Sweden; 3 Department of Molecular Medicine and Surgery, Karolinska Institutet, Stockholm, Sweden; 4 Center for Molecular Medicine, Karolinska Institutet, Stockholm, Sweden; 5 Center for Gender Medicine, Karolinska Institutet, Stockholm, Sweden; 6 Department of Clinical Sciences, Danderyd Hospital, Karolinska Institutet, Stockholm, Sweden; 7 Division of Surgery, Department of Clinical Science, Intervention and Technology, Karolinska Institutet, Stockholm, Sweden; 8 Division of Obstetrics and Gynecology, Department of Clinical Science, Intervention and Technology, Karolinska Institutet, Stockholm, Sweden; Duke University Medical Center, United States of America

## Abstract

Elevated systemic pentraxin 3 (PTX3) levels appear to be a powerful marker of inflammatory status and a superior outcome predictor in patients with chronic kidney disease (CKD). As previous data imply that PTX3 is involved in vascular pathology and that adipose tissue mass may influence circulating PTX3 levels, we aimed to study the importance of adipose tissue expression of *PTX3* in the uremic milieu and its relation to endothelial dysfunction parameters. Plasma PTX3 and abdominal subcutaneous adipose tissue (SAT) *PTX3* mRNA levels were quantified in 56 stage 5 CKD patients (median age 57 [range 25–75] years, 30 males) and 40 age and gender matched controls (median age 58 [range 20–79] years, 27 males). Associations between PTX3 measures and an extensive panel of clinical parameters, including surrogate markers of endothelial function, were assessed. Functional *ex vivo* studies on endothelial status and immunohistochemical staining for PTX3 were conducted in resistance subcutaneous arteries isolated from SAT. SAT *PTX3* mRNA expression correlated with plasma PTX3 concentrations (rho = 0.54, p = 0.0001) and was increased (3.7 [0.4–70.3] *vs.* 1.2 [0.2–49.3] RQ, p = 0.02) in CKD patients with cardiovascular disease (CVD), but was not significantly different between patients and controls. The association to CVD was lost after adjustments. SAT *PTX3* mRNA levels were independently correlated to asymmetric dimethylarginine and basal resistance artery tone developed after inhibition with nitric oxide synthase and cyclooxygenase (rho = −0.58, p = 0.002). Apparent positive PTX3 immunoreactivity was observed in both patient and control arteries. In conclusion, fat *PTX3* mRNA levels are associated with measures of endothelial cell function in patients with CKD. PTX3 may be involved in adipose tissue-orchestrated mechanisms that are restricted to the uremic milieu and modify inflammation and vascular complications in CKD patients.

## Introduction

Endothelial dysfunction is a common feature in the uremic milieu [1], which predicts cardiovascular events and poor outcome in patients with chronic kidney disease (CKD) [2]. Although CKD patients run a markedly increased cardiovascular disease (CVD) risk [3], these complications are often underdiagnosed and undertreated in this population. The presence of endothelial dysfunction is linked to inflammation [4], which has repeatedly been shown to enhance the risk for CVD mortality in both renal [5] and non-renal populations [6]. CKD patients seem to be particularly prone to develop persistent inflammation, potentially due to raised production as well as decreased renal clearance of pro-inflammatory cytokines [7].

The soluble pattern recognition receptor pentraxin-3 (PTX3; MW 40.2 kDa) belongs to the pentraxin superfamily, a group of evolutionary conserved acute-phase reactants which also includes C-reactive protein (CRP) (57% amino acid identity to CRP and serum amyloid P) [8]. PTX3 participates in the cellular cascades of the innate, antigen unspecific, humoral immunity. However, beyond its expanding importance as an inflammatory marker, PTX3 possesses numerous additional regulatory functions, including effects on angiogenesis, atherosclerotic lesion development, apoptopic cell clearance, tissue repair and regulation of renal immunopathology [9]. The synthesis of PTX3 is prompt and reaches a peak within 4–6 hours after the trigger signal, mainly activated by interleukin (IL)-1 beta, lipopolysaccharides, Toll-like receptor ligands and tumor necrosis factor (TNF) [10]. Although PTX3 is primarily produced by inflammatory cells, such as monocytes [11] and neutrophils [12], this long pentraxin is also produced by endothelial cells, fibroblasts [13], [14], smooth muscle cells [15], mesangial cells [16], and myeloid dendritic cells [17]. In addition, recent evidence suggests that also adipocytes express PTX3 [18], [19]. Thus, in contrast to short pentraxins (such as CRP), which are only expressed by hepatocytes, PTX3 is produced at the actual site of inflammation.

The finding that inflammatory stimuli dramatically increase the expression of PTX3 in vascular endothelial cells has drawn the attention to PTX3 as an important marker for vascular pathology [20]. Besides infectious diseases, serum PTX3 levels are upregulated in different clinical vascular and metabolic conditions, such as myocardial infarction [21], atherosclerosis [22], ischemic stroke [23], acute coronary syndromes [23], [24], vasculitis [25], coronary heart disease [26], rheumatoid arthritis [27], insulin resistance [28], obesity [29] as well as preeclampsia [30]. Analogous to other inflammatory biomarkers, systemic levels of PTX3 also increase as renal function declines and predict increased cardiovascular and overall mortality risk in CKD patients, independent of traditional risk factors [31], [32], [33].

Obesity is associated with a state of chronic, low-grade inflammation and macrophage infiltration [34]. Associations between total abdominal fat and inflammatory markers have also been documented in CKD [35], [36], [37], [38], [39], which may suggest that adipose tissue contributes to the inflamed uremic phenotype. For instance, increased fat mass is associated with elevated levels of pro-inflammatory IL-6 and CRP [35], [40], [41] and, in a longitudinal study, fat mass increments were associated with increasing levels of soluble CD163 (a protein expressed by macrophages) [42]. In addition, efforts to characterize adipose tissue of CKD patients have indicated that several pro-inflammatory genes are upregulated [43], [44] and CD68-postitive immunocompetent cells are increased in the uremic milieu [41], [43]. Recent studies have also demonstrated elevated adipose tissue expression of *PTX3* in obesity [19]. Paradoxically however, systemic PTX3 levels were reported to be inversely associated with increased fat mass in both CKD [40] and non-CKD [19], [45] patients.

Against this background we hypothesized that adipose tissue has a regulatory effect on systemic PTX3 concentrations in CKD, and that PTX3 expression in adipose tissue could affect local endothelial function. In an attempt to investigate this, we employed several study approaches. First, circulating PTX3 levels and adipose tissue expression of *PTX3* mRNA were analyzed and related to measures of body composition in a group of carefully phenotyped incident dialysis patients (CKD stage 5) and non-CKD controls. In addition, to study cell and depot-specific expression patterns, we compared *PTX3* expression between human subcutaneous (SAT) and visceral (VAT) fat mass as well as between isolated adipocytes and intact adipose tissue. Second, to explore the role of renal function *per se* on circulating PTX3 levels, we measured PTX3 in a group of CKD patients before and one year after renal transplantation. Finally, to gain more insight into the relation between PTX3 and endothelial function, *PTX3* expression was related both to surrogate circulating markers of endothelial injury and functional *ex vivo* data on small resistance arteries isolated from adipose tissue. Immunohistochemical staining for PTX3 was also conducted to compare the occurrence of PTX3 in arteries from CKD patients and controls. Altogether, we show that adipose tissue expression of *PTX3* is associated with circulating markers as well as functional data of endothelial function, but that the amount of fat mass is probably of less importance in mechanisms causing elevated systemic PTX3 levels in patients with CKD.

## Methods

### Ethics statement

The study protocol was undertaken in adherence with the Declaration of Helsinki and approved by the Regional Ethical Review Board at Karolinska Institutet in Stockholm. Each participant gave written informed consent.

### CKD-5 patients and controls

As a part of an ongoing prospective study on patients with late stage CKD (CKD stage 5) [46], 56 consecutive patients were recruited between 2004–2011 at the renal outpatient clinic at Karolinska University Hospital Huddinge, shortly before initiating dialysis (median glomerular filtration rate 7 (3–12) ml/min). The study exclusion criteria were age below 18 years or above 75 years, clinical evidence of acute infection, active vasculitis or liver disease at the time of inclusion, or unavailability of data and samples. In this patient cohort, the main causes of CKD were diabetic nephropathy (n = 16), polycystic kidney disease (n = 11) and chronic glomerulonephritis (n = 7). Twenty patients (37%) were previously reported to have a history of CVD and 21 patients (38%) were diagnosed with diabetes mellitus type 1 (n = 8) or type 2 (n = 13). Common medical treatments among the patients were beta-blockers (n = 31, 57%) and statins (n = 26, 48%), ACE- inhibitors (n = 43, 80%), calcium antagonists (n = 29, 54%) and diuretics (n = 46, 85%). A subgroup consisting of 28 patients (46% males, age 50 [26–69] years) that underwent renal transplantation were followed prospectively and follow-up data on plasma PTX3 and GFR were collected approximately one year after the transplantation.

The non-CKD control group (n = 40) included patients scheduled for elective hernia repair (n = 19) or laparoscopic cholecystectomy (n = 21) without known renal, cardiovascular or diabetic disease. Individuals were also excluded from the study if presenting signs of systemic inflammation prior to surgery or if data or samples were unavailable. A small proportion of the control subjects were on treatment with beta-blockers (n = 4), calcium antagonists (n = 5), ACE inhibitors (n = 4) and statins (n = 3). All control subjects were consecutively recruited at Karolinska University Hospital Huddinge between 2007–2011.

#### Clinical characteristics and anthropometric evaluation

Glomerular filtration rate (GFR) was estimated by taking the mean of urea and creatinine clearance (CKD-5 patients), or from the plasma level of cystatin C (non-CKD controls). Information on CVD, i.e. presence of ischemic cardiac disease, peripheral vascular disease and/or cerebrovascular disease, diabetes diagnosis and ongoing medications were obtained from the patients' medical records. Body mass index (BMI) was calculated by kg/m^2^ and Dual-energy X-ray Absorptiometry (DXA) evaluated body fat content.

#### Biochemical analyses and tissue sampling

Blood samples were drawn prior to the surgical procedure (after an overnight fast) from which plasma and serum were obtained. If not analyzed immediately, samples were stored at −70°C for later use. Conventional blood parameters such as glucose, creatinine, serum albumin, cholesterols, and high sensitivity CRP (hsCRP) were assessed by standard analytical methods at the Department of Clinical Chemistry, Karolinska University Hospital, Huddinge. Measurements of specific biochemical markers were performed using commercially available assays or enzymatic procedures: solid-phase, chemiluminescent immunometric assays (Immulite system, Siemens Healthcare Diagnostics Inc., Tarrytown, NY, USA) were used to measure IL6, C-peptide and TNF in serum; Quantikine Human Pentraxin 3/TSG-14 Immunoassay (R&D Systems, Inc., Minneapolis, MN, USA) was used to determine plasma PTX3; and an ADMA-Arginine ELISA (DLD Diagnostika GmbH, Hamburg, Germany) was performed to measure endogenous ADMA and L-arginine in plasma.

The SAT biopsies were collected in conjunction to peritoneal dialysis catheter insertion (CKD-5 patients), hernia repair (controls) or laparoscopic cholecystectomy (controls) after an overnight fast. Following incision of the skin of the lower abdominal region, approximately 1 g of SAT was obtained with sharp dissection from the anterior abdominal wall. The biopsies were immediately frozen and stored in −70°C. Concurrently, supplementary SAT biopsies were placed in cold PSS (physiological salt solution) for the immediate isolation of subcutaneous resistance arteries (internal diameter ≈200 µm), which was followed by either functional studies or storage in −70°C pending immunohistochemical analyses.

### Functional investigations of subcutaneous resistance arteries

Twenty-seven patients with CKD-5, in which *PTX3* mRNA expression was assessed, donated subcutaneous fat for functional studies of isolated arteries, as described elsewhere [1]. Briefly, resistance-size arteries were dissected from surrounding fat tissue and mounted on two stainless steel wires (25 µm in diameter) in the organ baths of a four-channel wire myograph (model 610, Danish Myo Technology; Aarhus, Denmark) as described previously [47]. Functional investigations included assesment of responses to endothelium-dependent agonists acethylcholine (ACh) and bradykinin (BK) before and after incubation with nitric oxide synthase (NOS) inhibitor (300 µmol/L) and cyclooxygenase (COX) inhibitor in order to determine the relative contribution of endothelium-derived factors that confer endothelium-dependent dilatation and contribute to the maintenance of basal tone. The level of increased resting tension of the arteries after incubation with L-NG-Nitroarginine Methyl Ester (L-NAME) together with Indo was considered as an index of vasoactive properties of the endothelium, reflecting a basal release of endothelium-derived factors such as nitric oxide and to a lesser extent, if any, prostanoids.

### Non-CKD adipose tissue biopsies

Abdominal SAT and VAT mRNA from 20 obese subjects without renal dysfunction were available from a previously described cohort [48]. These samples were collected from non-diabetic patients undergoing bariatric surgery after a 12 hour fast. At the start of surgery, a piece of subcutaneous fat was obtained from one of the laparoscopic port-hole incisions as well as from the greater omentum. Samples were immediately frozen in liquid nitrogen and kept frozen at −80°C until analysis. SAT for paired samples of isolated adipocytes and intact SAT was obtained by needle aspiration under local anesthesia from 14 healthy subjects after an overnight fast. This cohort as well as the procedure to isolate mature adipocytes has been described elsewhere [48].

### RNA extraction and mRNA measurements

The procedures of RNA extraction, cDNA synthesis and quantitative real-time PCR were performed with commercial kits and assays, and applying adequate quality controls. Briefly, total RNA was isolated from the frozen tissue using RNeasy Lipid Tissue Mini Kit with DNase I digestion (QIAGEN Sciences, Germantown, MD, USA). The RNA concentration and quality were evaluated with NanoDrop ND-1000 Spectrophotometer (NanoDrop Technologies Inc., Wilmington, DE, USA) and Agilent 2100 Bioanalyzer (Agilent Technologies Inc., Santa Clara, CA, USA) respectively, before reverse transcription into cDNA (QuantiTect Reverse Transcription Kit, QIAGEN GmbH, Hilden, Germany). mRNA levels of *PTX3*, *IL-6*, *TNF*, *CD68* and *MCP1* were assessed by means of real-time PCR quantification using Applied Biosystems TaqMan Gene Expression Assays targeted for PTX3 and endogenous control genes (Eukaryotic 18S rRNA, glucuronidase, beta [GUSB] and TATA box binding protein [TBP]). PCR reactions were run in triplicates with Applied Biosystems TaqMan Universal Master Mix No AmpErase UNG on an ABI 7900HT instrument (Life Technologies Corp., Carlsbad, CA, USA). The obtained threshold cycles (C_t_) were converted into relative gene expression quantities (RQ, arbitrary unit) by a modified delta-delta-C_t_-method, involving normalization against the three endogenous control genes [49].

### Immunohistochemistry

Crysections (8 µm thick) of subcutaneous arteries were mounted on Superfrost Plus slides (Thermo Scientific, Gerhard Menzel GmbH, Braunschweig, Germany) and kept at −80°C until required. Before immunohistochemical staining, the sections were thawed and post-fixed with pre-cooled methanol. The staining was conducted according to the protocol referred to UltraVision ONE Detection System HRP Polymer & DAB Plus Chromogen (Thermo Fisher Scientific, Chesire, UK). In brief, slides were pretreated with Hydrogen Peroxide Block followed by 15% normal goat serum (Abcam, Cambridge, UK) for 1 hour. The primary antibody, anti-PTX3, N-terminal antibody produced in rabbit (Sigma-Aldrich, Schnelldorf, Germany) was diluted 1:300 in PBS with 2.5% goat serum, applied to slides and incubated for 3 hours at 4°C. The primary antibody was omitted in the negative controls. The PTX3 binding was revealed using a universal secondary antibody polymer formulation conjugated to horseradish peroxidase (HRP). The HRP activity was subsequently visualized with diaminobenzidine (DAB) substrate/chromogen and counterstaining with hematoxylin was performed. Finally, slides were coverslipped using Aqua-Mount mounting media (Thermo Fisher Scientific CA, USA) and examined and photographed under light microscopy (DN100, E600, Nikom, Tokyo, Japan) with X20 and X40 objectives. Staining was performed on eight samples each of CKD-5 patients and controls (duplicate samples). Three independent observers that were blinded to the study groups semi-quantitatively assessed the immunohistochemistry staining intensity. A scale from 0 to 3 was used, were 0 corresponded to absence of staining and 3 to maximum staining intensity.

### Statistical analyses

Data generated in the study deviate from the normal distribution, as assessed by the Shapiro-Wilk test. Values are therefore expressed as median (range) and non-parametric statistical tests were applied. Comparisons of continuous variables between groups were performed with Wilcoxon rank sum test, Wilcoxon signed rank test and Kruskal-Wallis one-way analysis of variance as appropriate. Chi-square tests were used for comparisons of nominal variables between groups. Univariate correlations (rho) were evaluated with Spearman's rank correlation analysis, whereas a multivariate regression analysis was applied to assess associations between SAT *PTX3* mRNA levels and independent variables. The residuals of the multivariate analysis were normally distributed. Multivariate logistic regression models were used to investigate associations between CVD (presence/absence) and plasma PTX3 or SAT *PTX3* mRNA levels. The threshold for statistical significance (P) was set as <0.05. Analyses were performed using statistical software JMP 9.0.0 or SAS version 9.2 (SAS Institute Inc, Cary, NC).

## Results

### General characteristics of CKD-5 patients and non-CKD controls

Descriptive clinical data of CKD-5 patients and non-CKD controls are detailed in **Table 1**. CKD-5 patients and controls displayed similar age and gender distributions. As expected, whereas serum albumin levels were lower, TNF, triglycerides and C-peptide levels were significantly higher in the CKD-5 group. Inherent to the underlying causes of surgery (cholecystitis or hernia) of the non-CKD subjects, the median BMI was higher in the control group than in the CKD-5 patients.

**Table 1:** Demography and clinical characteristics of non-CKD controls and CKD stage 5 patients.

	Controls	CKD-5	p-value*
	n = 40	n = 56	
**Etiology of CKD (%)**			
Diabetic nephropathy	NA	29	NA
Polycystic kidney disease	NA	20	NA
Chronic glomerulonephritis	NA	13	NA
Other causes	NA	38	NA
**Comorbidities**			
Diabetes mellitus (n)	0	21	NA
Type 1 diabetes (n)	0	8	NA
Type 2 diabetes (n)	0	13	NA
Cardiovascular disease (n)	0	21	NA
**General characteristics**			
GFR (ml/min)	90 (54–90)	7 (3–12)	<0.0001
Age (years)	58 (20–79)	57 (25–75)	0.79
Sex (males)	27	30	0.20
**Body composition**			
Body mass index (kg/m^2^)	27.2 (21.2–39.2)	24.0 (13.3–31.8)	<0.0001
Total fat mass (kg)	NA	19.5 (6.2–32.6)	NA
Truncal fat mass (kg)	NA	10.4 (2.6–19.9)	NA
Lean body mass (kg)	NA	48.0 (28.2–72.7)	NA
**Metabolic markers**			
Serum albumin (g/l)	39 (31–43)	34 (21–47)	<0.0001
Cholesterol (mmol/l)	4.8 (2.7–7.6)	4.6 (0.2–9.8)	0.12
Triglycerides (mmol/l)	1.2 (0.5–2.3)	1.5 (0.6–5.6)	0.007
Plasma glucose (mmol/l)	5.4 (4.6–7.7)	5.0 (3.2–22)	0.15
HbA1c, IFCC units (mmol/mol)	36 (30–50)	37 (21–87)	0.57
C-peptide (ng/ml)	2.3 (0.5–7.8)	6.3 (0.1–19)	0.0001
**Endothelial function**			
ADMA (µmol/l)	0.6 (0.3–1.6)	0.9 (0.4–1.3)	<0.0001
Arginine/ADMA	168 (40–291)	135 (51–314)	0.02
**Inflammatory markers**			
PTX3 (ng/ml)	0.7 (0.1–2.3)	1.4 (0.4–7.5)	<0.0001
hsCRP (mg/l)	1.9 (0.4–42)	2.6 (0.2–55)	0.42
IL-6 (pg/ml)^1^	3.1 (1.3–18)	6.6 (0.8–32)	0.002
TNF (pg/ml)	6.5 (4.0–10)	14 (7.1–27)	<0.0001
**Fat mRNA expression (RQ)**			
PTX3	1.0 (0.3–30.1)	1.9 (0.2–70.3)	0.16
IL-6^3^	4.4 (1.0–90.4)	5.6 (1.4–29.5)	0.45
CD68	1.0 (0.02–5.7)	1.0 (0.1–3.1)	0.87
MCP1	0.6 (0.04–7.2)	0.5 (0.1–8.1)	0.90

Continuous data presented as mean (range). *Comparisons between groups with Wilcoxon rank sum test; ^1^Controls n = 22, patients n = 35; ^2^Controls n = 14, patients n = 11; ^3^Controls n = 22, patients n = 20. ADMA, asymmetric dimethylarginine; CKD, chronic kidney disease; GFR, glomerular filtration rate; hsCRP, high-sensitive C-reactive protein; IL-6, interleukin 6; MCP1, monocyte chemoattractant protein 1; NA, non applicable; PTX3, pentraxin 3; TNF, tumor necrosis factor.

### PTX3 in relation to clinical and biochemical variables in CKD-5 patients

**Figure 1:**
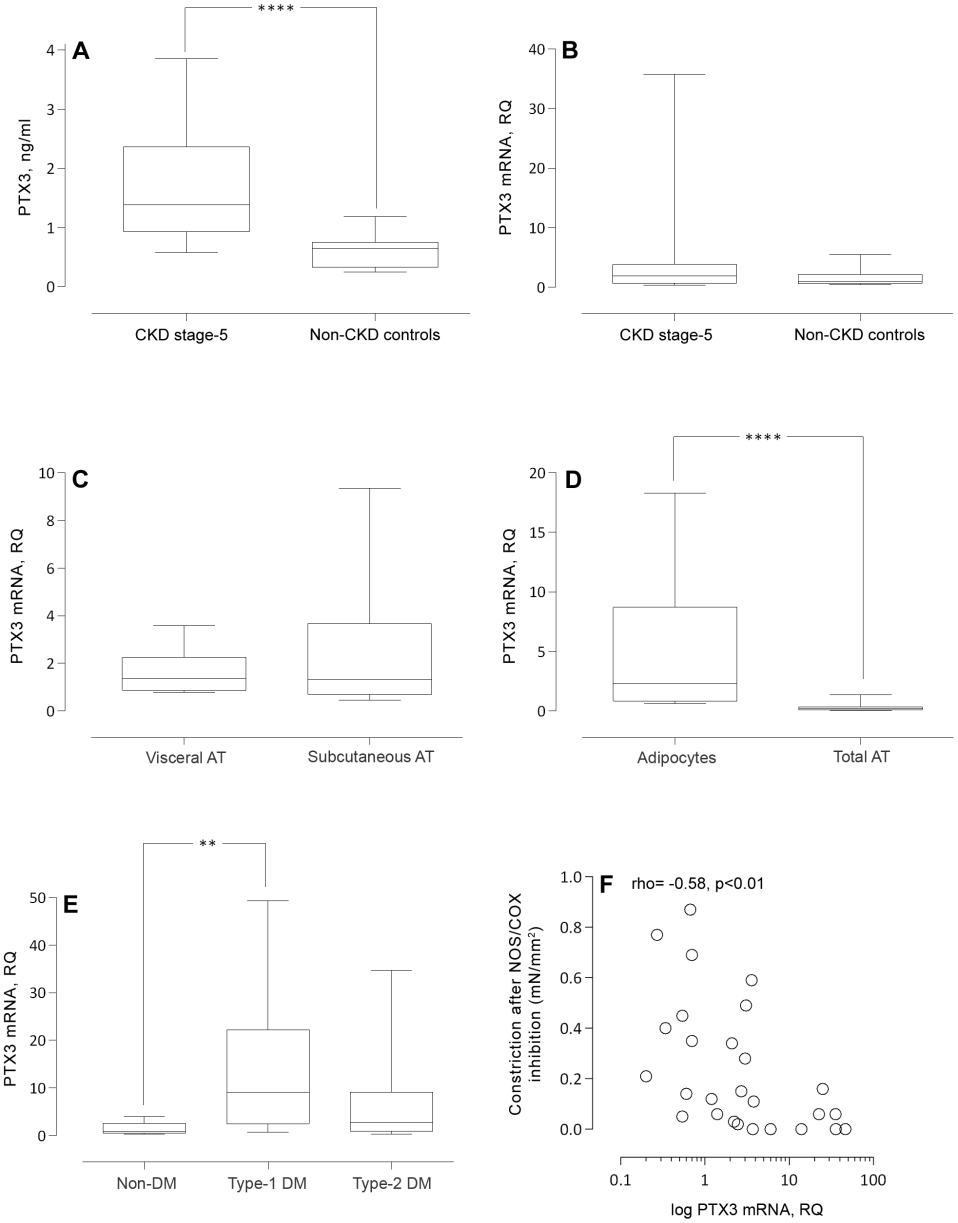
*PTX3* mRNA is expressed in isolated adipocytes and levels correlate with endothelial dysfunction in CKD patients. (A) Plasma PTX3 concentrations (ng/ml) measured in 39 non-CKD controls and 53 patients with CKD stage 5. *PTX3* mRNA quantities (relative quantity, RQ, arbitrary units) analysed in (B) abdominal subcutaneous adipose tissue of 40 non-CKD controls and 56 patients with CKD stage 5, (C) subcutaneous and visceral fat depots obtained from 20 non-CKD patients, (D) isolated adipocytes and intact adipose tissue (n = 14) as well as in (E) patients with CKD stage 5 and no diabetes mellitus (n = 35), diabetes mellitus type 1 (n = 8) or diabetes mellitus type 2 (n = 13). (F) Negative correlation (Spearman's rank) between subcutaneous adipose tissue *PTX3* mRNA quantities (log transformed) and constriction (basal tone) after nitric oxide synthase (NOS) and cyclooxygenase (COX) inhibition in 27 patients with CKD stage 5. Statistical differences between groups were evaluated with Wilcoxon rank sum test (A–B), Wilcoxon signed rank test (C–D) and Kruskal-Wallis one-way analysis of variance (E). **, p<0.01; ***, p<0.001; ****, p<0.0001.

Median plasma PTX3 protein level was significantly higher in patients than in controls (1.4 [0.4–7.5] *vs.* 0.7 [0.1–2.2] ng/ml, p<0.0001, **Figure 1A**). *PTX3* mRNA was expressed in both patient and control SAT but no significant differences were observed (1.9 [0.2–70.3] *vs*. 1.0 [0.3–30.1] RQ, p = 0.16, **Figure 1B**). Univariate analyses showed that plasma PTX3 and *PTX3* mRNA expression were positively correlated in patients (**Table 2**), but not in the non-CKD controls (data not shown). In a multivariate model, plasma PTX3 was a significant predictor of *PTX3* mRNA independent of age, sex and diabetes (**Table 3**). Circulating IL-6 concentrations, but not TNF and CRP, were positively correlated to plasma PTX3 (**Table 2**). Uremic patients with a medical history of CVD had a significantly higher circulating median PTX3 (1.8 [0.5–7.5] *vs.* 1.1 [0.4–5.9] ng/ml, p = 0.02) and SAT *PTX3* mRNA levels (3.7 [0.4–70.0] *vs.* 1.2 [0.2–49.3] RQ, p = 0.02) compared to patients without CVD events. When adjusting for predictors of CVD (age, sex, diabetes mellitus) as well as ADMA levels and Arginine/ADMA ratio in multivariate logistic regression models this association was lost (**Table 4**).

**Table 2:** Univariate Spearman correlations with circulating PTX3 (A) or adipose tissue *PTX3* mRNA (B) and clinical/biochemical variables in 56 patients with CKD stage 5.

	A		B	
Variables	Plasma PTX3, ng/ml		PTX3 mRNA, RQ	
	rho	p-value	rho	p-value
**General characteristics**				
GFR (ml/min)	−0.19	0.24	0.15	0.34
Age (years)	0.13	0.33	0.11	0.41
**Body composition**				
Body mass index (kg/m^2^)	−0.02	0.90	0.22	0.11
Total fat mass (kg)	−0.21	0.16	−0.17	0.26
Truncal fat mass (kg)	−0.26	0.09	−0.15	0.30
Lean body mass (kg)	−0.01	0.99	0.21	0.15
**Metabolic markers**				
Serum albumin (g/l)	−0.25	0.08	−**0.39**	**0.004**
Cholesterol (mmol/l)	−0.10	0.50	−0.18	0.19
Triglycerides (mmol/l)	0.03	0.84	−0.15	0.26
Plasma glucose (mmol/l)	0.01	0.97	−0.03	0.82
HbA1c, IFCC (mmol/mol)	0.05	0.73	0.21	0.12
C-peptide (ng/ml)	−0.11	0.45	−**0.33**	**0.02**
**Endothelial function**				
ADMA (µmol/l)	0.11	0.27	**0.37**	**0.006**
Arginine/ADMA	−**0.34**	**0.01**	−**0.27**	**0.05**
**Inflammatory markers**				
hsCRP(mg/l)	−0.07	0.62	−0.04	0.78
IL-6 (pg/ml)	**0.33**	**0.03**	0.29	0.06
TNF (pg/ml)	0.18	0.21	0.14	0.30
**Fat mRNA expression (RQ)**				
PTX3	**0.54**	**0.0001**	NA	NA
IL-6	0.35	0.32	−0.26	0.43
TNF	0.10	0.86	−0.17	0.74
CD68	**0.33**	**0.02**	0.09	0.53
MCP1	0.25	0.08	**0.48**	**0.0004**

ADMA, asymmetric dimethylarginine; CKD, chronic kidney disease; GFR, glomerular filtration rate; hsCRP, high-sensitivity C-reactive protein; IL6, interleukin 6; MCP1, monocyte chemoattractant protein 1; NA, non applicable; PTX3, pentraxin 3; RQ, gene expression relative quantity; TNF, tumor necrosis factor.

**Table 3:** Multiple regression model predicting adipose tissue *PTX3* mRNA in 56 patients with CKD stage 5.

Adjusted r^2^ = 0.27; p = 0.002			
Variable	Beta	Standard Error	p-value
Age	−0.27	0.3	**0.04**
Sex (reference: male)	0.05	6.2	0.67
Diabetes mellitus (ref: no diabetes)			
Type 1 diabetes mellitus	−0.06	8.9	0.63
Type 2 diabetes mellitus	0.08	8.6	0.59
PTX3 (ng/ml) 1-SD increase	0.31	3.3	**0.02**
ADMA (µmol/l) 1-SD increase	0.36	7.9	**0.004**

ADMA, asymmetric dimethylarginine; CKD, chronic kidney disease; PTX3, pentraxin 3. Variables PTX3 and ADMA were expressed as 1-standard deviation (1-SD) increase.

**Table 4:** Multivariate logistic regression models investigating predictors of CVD (presence/absence) in 56 patients with CKD stage 5, including plasma PTX3 concentrations and Arginine/ADMA ratio (Model 1; pseudo R^2^ = 0.20, p = 0.0125) or adipose tissue *PTX3* mRNA and ADMA (Model 2; pseudo R^2^ = 0.16, p = 0.0547) as covariates.

	Odds ratio	95% Confidence Interval	p-value
**Model 1:**			
Plasma PTX3 (ng/ml)	1.34	0.77–2.35	0.305
Age (years)	1.05	0.99–1.12	0.094
Sex (reference: male)	0.45	0.13–1.66	0.233
Diabetes mellitus (reference: absence)	4.55	1.23–16.84	0.023
Arginine/ADMA	1.00	0.99–1.01	0.777
**Model 2:**			
PTX3 mRNA (RQ)	1.01	0.98–1.05	0.498
Age (years)	1.05	0.99–1.12	0.093
Sex (reference: male)	0.53	0.14–1.98	0.345
Diabetes mellitus (reference: absence)	4.75	1.29–17.46	0.019
ADMA (µmol/l)	0.85	0.04–17–19	0.917

ADMA, asymmetric dimethylarginine; PTX3, pentraxin 3; RQ, gene expression relative quantity.

### PTX3 levels after renal transplantation

**Figure 2:**
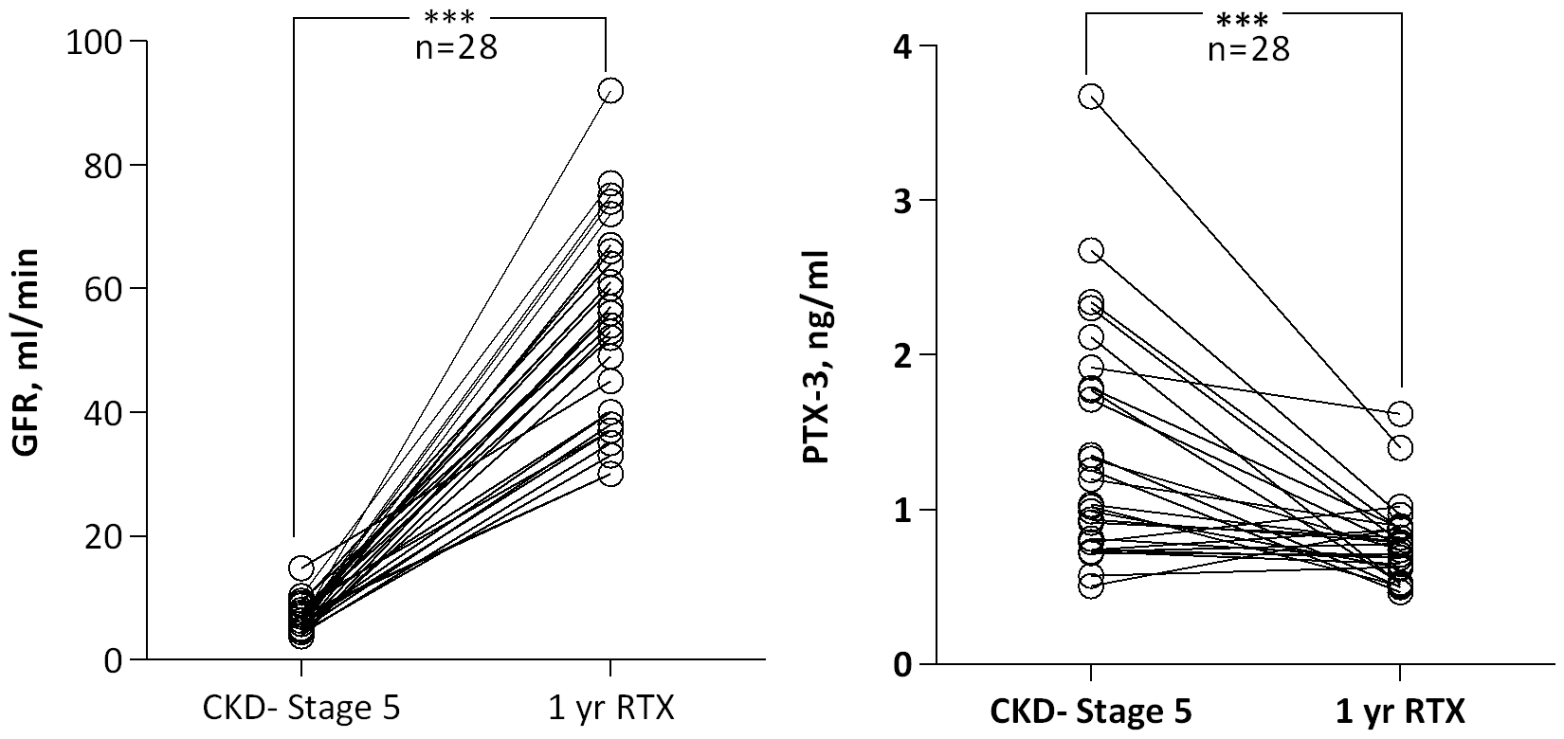
Plasma levels of PTX3 are decreased after kidney transplantation (RTx). One year after transplantation glomerular filtration rate (GFR) is improved and plasma PTX3 concentrations are significantly decreased compared to baseline (N = 28, Wilcoxon signed rank test). ***, p<0.001.

In a subgroup of CKD-5 patients, paired data was collected prior to start of dialysis and one year following renal transplantation. **Figure 2** shows significantly (p<0.0001) reduced circulating PTX3 concentrations (1.0 [0.5–3.7] to 0.8 [0.5–1.6] ng/ml) following transplantation. Although the decrease in PTX3 was paralleled by an improvement in GFR (6 [4]–[14] to 55 [30–92] ml/min, p<0.0001) delta PTX3 was not correlated to delta GFR (data not shown).

### PTX3 in relation to body composition and features of adipose tissue

Plasma PTX3 and SAT *PTX3* mRNA levels were inversely correlated to serum albumin but did not correlate to BMI or measurements on body fat mass in patients with CKD stage 5. Similarly, in the control group, there were no significant correlations between BMI and *PTX3* mRNA or PTX3 plasma concentrations. A strong positive correlation was demonstrated between SAT mRNA levels of *PTX3* and monocyte chemoattractant protein-1 (*MCP1*) (**Table 2**). Whereas *PTX3* mRNA expression did not differ between VAT and SAT (1.4 [0.6–9.34] *vs.* 1.3 [0.3–24.2] RQ, p = 0.37) (**Figure 1C**) the expression was higher (p = 0.0001) in isolated adipocytes (2.3 [0.6–20.4] RQ) compared to total adipose tissue (0.2 [0.04–2.3] RQ) (**Figure 1D**).

### PTX3 in relation to diabetes status in CKD-5 patients

CKD-5 patients were further evaluated according to their type of diabetes. As expected, type 1 diabetic patients had longer diabetes duration than type 2 diabetic patients (28 [18]–[51] *vs.* 12 [3]–[24] years, p = 0.0007) and C-peptide level below the detection limit (0.1 [0.1–0.1] *vs.* 2.6 [0.5–16.3] ng/ml; p<0.0003). Non-diabetic CKD-5 patients had a median C-peptide of 6.8 [3.0–18.6] ng/ml. Type 2 diabetic patients were older (63 [42–75] *vs*. 50 [31–63] years, p = 0.009) than patients with type 1 diabetes. The median HbA1c (IFCC) value differed markedly (p<0.0001) between the three groups with type 1 diabetic patients displaying a higher median HbA1c (56 [42–83] mmol/l) than both type 2 diabetic patients (38 [30–87] mmol/l) and non-diabetics (35 [21]–[44] mmol/l). The type 2 diabetic patients had a significantly (p = 0.001) higher frequency of CVD events (77%) than type 1 diabetic (50%) and non-diabetic (20%) patients. Also, plasma PTX3 levels were slightly higher (p = 0.04) in type 2 diabetic patients (1.8 [1.0–7.5] ng/ml] than in type 1 diabetic (1.5 [0.9–5.9] ng/ml) and non-diabetic patients (1.1 [0.4–4.0] ng/ml). On the other hand, type 1 diabetic patients had an increased median SAT *PTX3* mRNA level compared to type 2 diabetic and non-diabetic CKD patients (9.1 [0.7–49.3] *vs.* 3.7 [0.2–46.6] *vs.* 1.0 [0.2–70.3] RQ, p = 0.005), **Figure 1E**). However, multivariate regression analysis, including age, sex and PTX3 plasma levels, showed no independent association between diabetes status and SAT *PTX3* mRNA expression (**Table 3**).

### PTX3 in relation to ADMA and *ex vivo* endothelial function in CKD-5 patients

**Figure 3:**
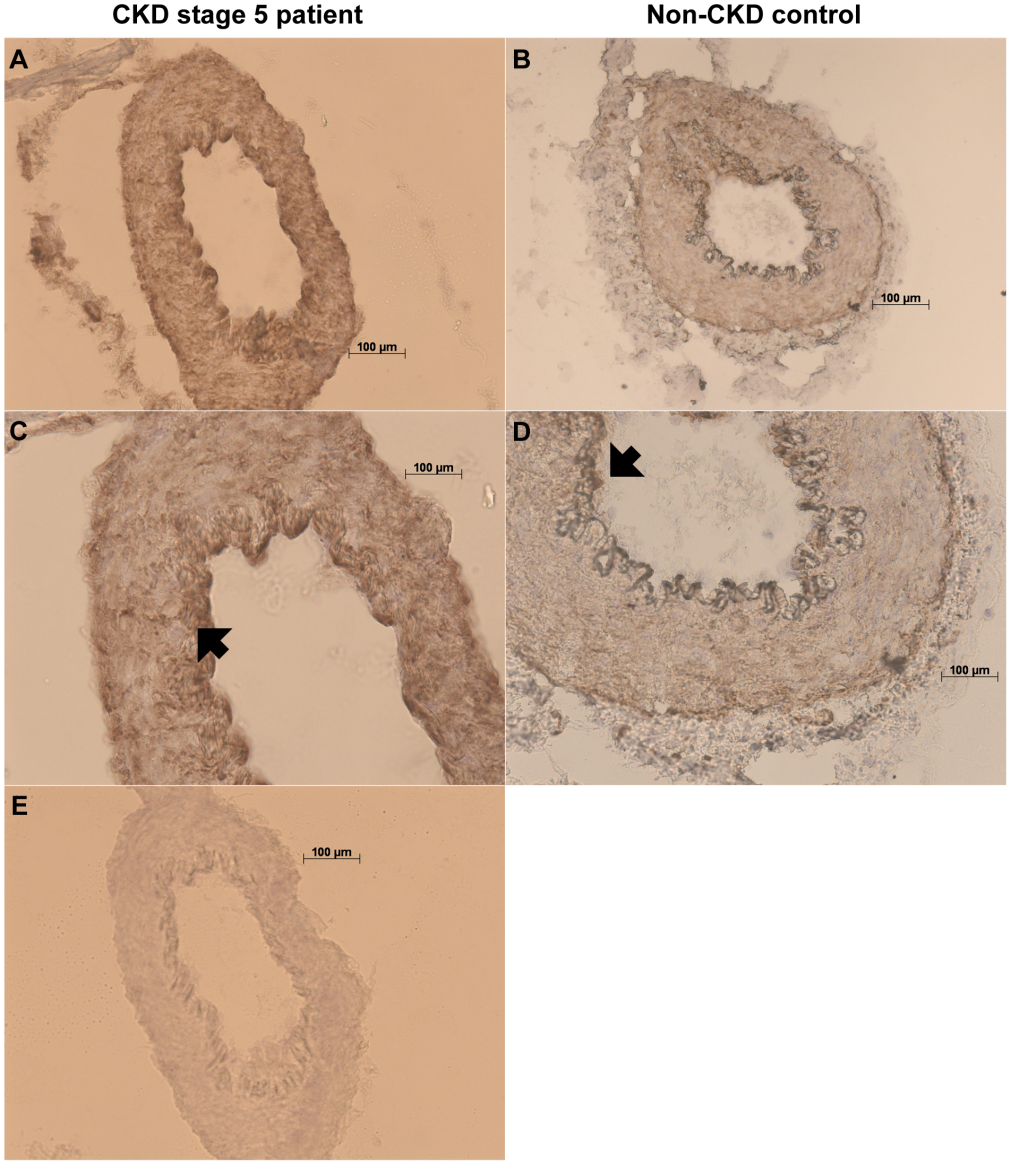
Positive immunohistochemical staining of PTX3 in resistance subcutaneous arteries from CKD stage 5 patients. Positive staining (brown) is shown in representative biopsies of CKD-5 patients (A, C) and non-CKD controls (B, D). No immunoreactivity was observed when anti-PTX3 antibody was absent (E). The PTX3-immunoreactivity is abundant in arteries, especially in endothelial cells of tunica intima, indicated by black arrowheads, both in patients (C) and controls (D). The slides were counterstained with hematoxylin. Images are magnified x10 (B), x20 (A, D, E) or x40 (C); scale bar 100 µm.

Circulating asymmetric dimethylarginine (ADMA) and the Arginine/ADMA ratio were used as surrogate biomarkers of endothelial dysfunction. Both plasma PTX3 and SAT *PTX3* mRNA levels correlated negatively to Arginine/ADMA ratio, whereas circulating ADMA correlated positively to *PTX3* mRNA levels in Spearman rank correlation analyses (**Table 2**). Moreover, ADMA was identified as a significant predictor of SAT *PTX3* mRNA expression, independent of age, sex, DM and plasma PTX3 (**Table 3**). No associations between plasma PTX3, SAT *PTX3* mRNA, ADMA levels AND Arginine/ADMA ratio were observed in the non-CKD group (data not shown). Functional studies on isolated arteries from a subset of CKD-5 patients and non-CKD controls demonstrated a negative correlation (rho = −0.58, p = 0.002) between SAT *PTX3* mRNA expression and basal tone after NOS/COX inhibition (**Figure 1F**) in patients but not in controls. However, there was no correlation between basal tone after NOS and COX inhibition and mRNA expression in fat tissue for other inflammatory markers tested; i.e. IL-6, TNF, CD68 and MCP1 (data not shown). Immunostaining revealed abundant PTX3 immunoreactivity in the vascular wall, possibly with a predominance in endothelial cells, of subcutaneous arteries of CKD-5 patients (**Figure 3**). However, the PTX3 immunoreactivity intensity in patient arteries did not differ from that of control subjects (2.3 [1.3–3.0] *vs*. 2.0 [1.0–3.0] a.u., p = 0.13).

## Discussion

We report that only patients with CKD show an association between SAT *PTX3* mRNA and plasma PTX3 levels, despite similar SAT *PTX3* mRNA levels in patients and non-CKD controls. Moreover, the observed associations, positive and inverse respectively, between SAT *PTX3* mRNA levels and elevated ADMA levels (adjusted for age, sex and diabetes mellitus) and *ex vivo* resistance artery constriction, were also restricted to CKD. We also reveal distinct immunopositivity for PTX3 in both CKD and non-CKD arteries. Although the present study demonstrated that a clinical history of CVD conferred higher circulating PTX3 and SAT *PTX3* mRNA levels in CKD patients, these associations were lost after adjusting for age, sex, diabetes and ADMA (or Arginine/ADMA). *PTX3* mRNA levels were higher in isolated adipocytes compared to intact adipose tissue but did not differ between VAT and SAT and the present results did not confirm an association between PTX3 and total or truncal (visceral) body fat mass. Finally, we report that circulating levels of PTX3 decrease following renal transplantation. Taken together, although our findings imply that reduced renal clearance may largely account for the marked increases in circulating PTX3 concentrations, we show that adipose tissue expression of *PTX3* is associated with PTX3 plasma protein levels and other circulating markers, as well as functional data of endothelial function. This suggests a role for uremic adipose tissue as an intermediate node in PTX3-pathways triggering endothelial aberrations and CVD.

Characterized as an innate immunity protein, PTX3 regulates not only inflammatory responses but is involved in a range of important biological mechanisms, including vascular pathology [20]. Several studies have shown that plasma PTX3 concentrations are elevated in CKD, and that increased circulating levels of PTX3 predict higher risk for all-cause and cardiovascular mortality [32], [33], [50], [51]. This is also observed in other patient groups, such as in patients with coronary heart disease [26], ischemic stroke [23] and acute chest pain [52], where high levels of PTX3 predict poor outcome. Following an appropriate trigger signal, PTX3 synthesis occurs in various cells and peripheral tissues at the actual site of inflammation [8]. So far, it has not been clear to what extent the complex and pro-inflammatory uremic milieu influences PTX3 levels and local PTX3 tissue production. As suggested by previous observational data from our group, showing associations between fat mass and plasma levels of PTX3 in CKD patients [40], as well as the emerging literature on plausible pathophysiological interactions between fat and inflammatory modulators in CKD [35], [36], [37], [38], [39], adipose tissue may represent a promising target for studies on local PTX3 expression. Adipocyte expression of PTX3 in humans has recently been demonstrated [18], [19], but not in patients with CKD. Indeed, we found that *PTX3* mRNA was ubiquitously expressed in adipose tissue from the CKD patients and positively associated with plasma PTX3 concentrations, but to our surprise, measures on fat mass associated neither with mRNA nor plasma levels of PTX3. Moreover, our analyses revealed no significant differences in the adipose tissue *PTX3* mRNA levels between the CKD-5 patients and non-CKD controls, indicating no obvious uremic-related abnormalities in the adipose tissue expression levels of *PTX3*. Instead, our results from transplanted patients support the notion that reduced clearance is a pivotal factor contributing to the elevated systemic PTX3 levels when renal function declines. However, as this analysis was only conducted on a limited number of patients and as PTX3 actually increased in some patients after renal transplantation (Figure 2), an increase in local PTX3 generation, by adipose tissue as well as other organs and cells, may not be ruled out. Furthermore, we cannot discard the possibility that normalization of circulating PTX3 after transplantation may, in addition to reversal of uremia, be secondary to immunosuppressive therapy. Studies on larger renal cohorts with comprehensive PTX3 tissue expression data may help to clarify the implication of our observations.

The pathophysiological role for PTX3 in adipose tissue is not well studied but our results agree with previous reports on PTX3 as a marker for vascular pathology [20]. For instance, we demonstrate a strong positive correlation between SAT expression of *PTX3* and *MCP1*. MCP1 is released under inflammatory conditions and seems to play a role in vascular endothelial dysfunction; it is highly expressed in human atherosclerotic lesions and drives monocyte recruitment into the arterial wall and developing lesions [53]. We also show independent and strong associations between *PTX3* mRNA expression and ADMA levels. Recent studies by our group suggest that raised ADMA plasma levels are indicative of endothelial dysfunction in CKD patients, linked to atherosclerosis progression and enhanced vascular resistance [1], [54]. Our finding corroborates previously reported associations between elevated PTX3 and surrogate markers of endothelial dysfunction. For instance, elevated plasma levels of PTX3 associate to VCAM-1 [33], flow-mediated dilatation [50] and cardiovascular outcomes in CKD patients [51], as well as with altered endothelial function during preeclampsia [30]. Conversely, marked decreases in PTX3 levels were associated with decreased albuminuria and improved flow-mediated dilatation following a 12 week intervention with ACE-inhibitors (ramipril) in type 2 diabetic patients [55]. Furthermore, our finding that CKD patients with CVD had both higher adipose tissue *PTX3* mRNA and plasma PTX3 levels is in line with a recent study in humans demonstrating a relationship between higher PTX3 levels and decreased arterial distensibility in obesity [29] and indirectly supports potential links between adipose tissue, PTX3 and endothelial dysfunction. This relation is further strengthened by our novel functional *ex vivo* data demonstrating a negative correlation between the basal tone (after NOS/COX inhibition) and *PTX3* mRNA expression in SAT as well as the abundant PTX3 immunopositivity in the endothelium of subcutaneous arteries. Interestingly, the absence of correlations between basal vascular tone and other inflammatory markers; i.e. IL-6, TNF, CD68 and MCP1, suggests that PTX3 *per se* plays a unique role in the regulation of endothelial function. Also, the notion that PTX3 only weakly correlated to these inflammatory molecules indicates that increased PTX3 may reflect endothelial function rather than systemic inflammation in the uremic milieu.

Although elevated PTX3 levels consistently predict poor outcome in several different patient groups and many studies suggest that elevated PTX3 is a sensitive marker of inflammatory endothelial activation, some data in mice suggest that PTX3 may have atheroprotective functions. Norata *et al* [56] demonstrated that deficiency of PTX3 is associated with increased heart damage and increased inflammatory response in a model of acute myocardial infarction (caused by coronary artery ligation). In accordance, Salio *et al* [57] demonstrated a cardioprotective role of PTX3 in acute myocardial infarction in mice. These animal experiments are supported by a recent study showing that circulating endothelial progenitor cells (which promote vascular repair and vasculogenesis) correlate with PTX3 in patients with peripheral artery disease [58]. Taken together, although elevated PTX3 is consistently linked to endothelial dysfunction and CVD, it is still elusive whether PTX3 is a non-causative maladaptive marker of endothelial inflammation or if it is actually causally related, either actively promoting the development of endothelial dysfunction, or providing protection and controlling the damage of the inflamed endothelium.

PTX3 is an intriguing inflammatory molecule and although much remains unsolved, our results add knowledge on its local expression and relationship to CKD phenotypes. A main strength of our study is the detailed clinical characterization of the study participants and, to the best of our knowledge, no previous study has reported an association between adipose tissue *PTX3* expression and functional data on endothelial parameters in the uremic milieu. However, the results of the present study should be interpreted in the light of some limitations. First, we would like to acknowledge the fact that gene expression might not truly reflect protein synthesis and, moreover, given the limited number of study subjects as well as the observational study design, the findings of the present study need further confirmatory analyses, e.g. adipose tissue immunostaining and analyses of fat cell secretion of PTX3. Complementary studies, such as investigations PTX3 expression in endothelial and inflammatory cells as well as other fat depots would also be of interest. Preliminary data in this study show that isolated adipocytes express *PTX3* mRNA (Figure 1D) to a higher extent than intact adipose tissue (including stromal and inflammatory cells) and, thus, it is plausible that mainly adipocytes contribute to the observed adipose tissue PTX3 content, with a smaller contribution from immune cells such as macrophages. Additionally, we could not show any differences in *PTX3* mRNA expression comparing non-CKD SAT and VAT, the two main fat depots in humans, suggesting no depot-specific expression patterns. Finally, inherent to the underlying causes of surgery in the non-CKD controls, BMI was higher in this group and we cannot exclude that this may have had an impact on the adipose tissue PTX3 expression data. However, we find this possibility unlikely as exclusion of the non-CKD controls with the highest BMI did not affect the results. Moreover, since diabetic subjects were not excluded from the CKD-5 cohort we stratified the patients according to diabetic status. In this context it is worth noting that the human PTX3 gene is located in a chromosomal region (chromosome 3, band q25 [13]) that is identified as a major locus for susceptibility to diabetic nephropathy in both type 1 and type 2 diabetes [59], [60]. Although significantly higher levels of SAT *PTX3* mRNA were found in fat from type 1 diabetic compared to type 2 diabetic and non-diabetic patients, this correlation was lost when adjusting for confounding variables (Table 3). Nevertheless, further research on *PTX3* expression and diabetic phenotypes in larger groups of diabetic patients matched for diabetes duration, age and sex may still be motivated.

In conclusion, this study shows that increased SAT *PTX3* mRNA expression is associated with increased circulating PTX3, higher ADMA levels and endothelial dysfunction in the uremic milieu. Our data support further investigations of adipose tissue-expressed PTX3 in mechanisms that modify inflammation and vascular function in CKD patients.
